#  Hurdles for the Delivery of Multinational Randomized Clinical Trials

**DOI:** 10.1001/jamanetworkopen.2025.18503

**Published:** 2025-07-02

**Authors:** Denise van Hout, Paul Mouncey, David Harrison, Marc Bonten, Lennie Derde, and the REMAP-CAP European Regional Investigators, Derek C. Angus, Aisha Anjum, Djillali Annane, Janis Best-Lane, Frank Brunkhorst, Maurizio Cecconi, Stephan Ehrmann, Anthony Gordon, Leanne Marie Hays, Esmee Kester, Niamh Mahon, Colin McArthur, Alistair Nichol, Svenja Peters, Sara Pugliese, Kathryn Rowan, Julian Torre-Cisneros, Sebastian Weis

**Affiliations:** 1 Julius Center for Health Sciences and Primary Care, University Medical Center Utrecht, Utrecht, the Netherlands; 2 Intensive Care National Audit and Research Centre, London, United Kingdom; 3 European Clinical Research Alliance on Infectious Diseases, Utrecht, the Netherlands; 4 Department of Intensive Care Medicine, University Medical Center Utrecht, Utrecht, the Netherlands; 5 Critical Care Medicine, University of Pittsburgh School of Medicine, Pittsburgh, Pennsylvania; 6 Division of Anaesthetics, Pain Medicine, and Intensive Care, Imperial College London, London, United Kingdom; 7 Prometheus University Hospital Institute Sepsis Comprehensive Center, Raymond Poincaré, Hospital Assistance Publique-Hôpitaux de Paris, University of Paris Saclay, Garches, France; 8 Center for Sepsis Control and Care, Department of Anesthesiology and Intensive Care Medicine, Jena University Hospital, Jena, Germany; 9 Department of Anesthesia and Intensive Care Units, Istituto di Ricerca e Cura a Carattere Scientifico, Humanitas Research Hospital, Rozzano, Milan, Italy; 10 Centre d’Étude des Pathologies Respiratoires, INSERM U1100, Tours, France; 11 Médecine Intensive Réanimation, INSERM CIC 1415, Critical Infections, Sepsis and Severe Disease Network, French Clinical Research Infrastructure Network, Centre Hospitalier Régional Universitaire de Tours, Tours, France; 12 University College Dublin-Clinical Research Centre, St Vincents University Hospital, Dublin, Ireland; 13 European Clinical Research Alliance on Infectious Diseases (Ecraid), Utrecht, the Netherlands; 14 Auckland City Hospital, Auckland, New Zealand; 15 Infectious Diseases Service, Reina Sofia University Hospital, Instituto Maimonides de Investigación Biomédica de Córdoba, University of Córdoba, CIBERINFEC, Córdoba, Spain; 16 Institute for Infectious Disease and Infection Control, Jena University Hospital, Friedrich-Schiller-University, Jena, Germany; 17 Department of Anesthesiology and Intensive Care Medicine, Jena University Hospital, Friedrich-Schiller-University, Jena, Germany; 18 Leibniz Institute for Natural Product Research and Infection Biology, Jena, Germany

## Abstract

**Importance:**

Ethical, administrative, regulatory, and logistical (EARL) procedures can hamper clinical trial delivery. Quantification of these hurdles is rare, prohibiting identification of areas for improvement.

**Objective:**

To identify and quantify EARL hurdles in trial delivery before and during the COVID-19 pandemic.

**Design, Setting, and Participants:**

This cohort study used data from the ongoing Randomized Embedded Multifactorial Adaptive Platform Trial for Community-Acquired Pneumonia to enable comparison of EARL procedures for multiple protocols across 19 European countries in the pre–COVID-19 pandemic (February 19, 2016 to March 10, 2020) and COVID-19 pandemic (March 11, 2020, to May 4, 2023) periods. Data were analyzed from November 2024 to March 2025 with contracts and protocol submissions as the units of analysis.

**Main Outcome and Measures:**

Time to (1) site contract completion, (2) regulatory and ethical approval (TTA), and (3) first patient in (FPI). The UK was compared with non-UK countries because of its distinct research infrastructure.

**Results:**

There were 257 fully signed first contracts with study sites for analysis. In the UK, contract completion times decreased by 97% (95% CI, 95% to 98%), from a median (IQR) of 196 (154 to 250) days in the pre–COVID-19 pandemic period to 5 (1 to 11) days during the COVID-19 pandemic. In non-UK countries, median (IQR) contract completion times were 224 (119 to 412) days and 183 (62 to 291) days before and during the COVID-19 pandemic, respectively (relative difference, −18%; 95% CI, −43% to 52%). In total, 44 interventions in 16 domains were submitted, yielding 232 protocol approvals for analysis. During the COVID-19 pandemic, median (IQR) TTA was 8 (5 to 31) days in the UK and 115 (47 to 103) days in non-UK countries (median difference, 107 days; 95% CI, 76 to 123 days), with large variation across non-UK countries. Time between approval and FPI during the COVID-19 pandemic was, on average, 3 months faster in the UK compared with non-UK countries (median difference, 90 days; 95% CI, 42 to 141 days).

**Conclusions and Relevance:**

This study found that EARL procedures were lengthy and variable between countries, reflecting different interpretations of trial regulations, with faster processes in the UK. These findings underscore the need to streamline processes across European countries to improve trial efficiency, in particular during future public health emergencies such as pandemics.

## Key Points

**Question:**

What are ethical, administrative, regulatory, and logistical (EARL) hurdles for the delivery of multinational randomized clinical trials (RCTs), and can these be quantified?

**Findings:**

This cohort study used data of the ongoing Randomized Embedded Multifactorial Adaptive Platform Trial for Community-Acquired Pneumonia, which allowed for comparisons of EARL procedures for 257 fully signed first contracts with study sites and 232 protocol submissions across 19 countries in the European region. Multiple EARL hurdles were identified, such as prolonged site contracting durations and time to approval, with high variability between countries.

**Meaning:**

These findings suggest that EARL hurdles compromise the ability to efficiently deliver RCTs, demonstrating the urgent need to overcome these hurdles, especially during public health emergencies like pandemics.

## Teaser

This cohort study quantifies ethical, administrative, regulatory, and logistical procedures for randomized clinical trials in Europe and identifies ways in which these procedures affected trial delivery before and during the COVID-19 pandemic.

## Introduction

Randomized clinical trials (RCTs) provide the highest level of evidence to inform medical practice. However, delivering RCTs presents substantial operational challenges that may jeopardize the success of the clinical trial enterprise.^1^

To protect participants and provide robust and valid results, trials must adhere to ethical, administrative, regulatory, and logistical (EARL) procedures. While these procedures are essential for ensuring patient safety and data integrity, they can also delay study start-up and consume substantial resources. Quantification of the burden of EARL procedures may facilitate efforts to optimize trial initiation by creating more fit-for-purpose and harmonized procedures across countries. However, quantifying the burden of these processes is difficult because data cannot easily be compared between different trials. The Randomized Embedded Multifactorial Adaptive Platform Trial for Community-Acquired Pneumonia (REMAP-CAP) is a multinational adaptive platform trial, where each new domain or intervention added to the trial is introduced through a new protocol element and submitted to regulatory authorities as a substantial amendment. Investigating varying types of interventions across many sites and countries with identical protocol elements, the trial provides a unique opportunity to compare EARL procedures in different settings.

During the COVID-19 pandemic, REMAP-CAP adapted to evaluate treatments for COVID-19 and was formally prioritized by the government and key health agencies in the UK, implementing several fast-track procedures.^2^ Other countries also prioritized COVID-19 research, but it is currently unknown whether these adaptations led to faster initiation of trials and interventions. The large number of new domains introduced in REMAP-CAP during this period and the major expansion of new trial sites enables comparisons between pre–COVID-19 pandemic and COVID-19 pandemic timelines, as well as between countries.

In this study, durations of key EARL procedures in the European region of REMAP-CAP were quantified for the pre–COVID-19 pandemic (2016-2020) and COVID-19 pandemic (2020-2023) periods, focusing on site contract completion times, time to regulatory and ethical approval (TTA), and time to first patient in (FPI), and comparing the UK with non-UK European countries. Using this empirical example, we aim to provide insights into where these processes can be optimized to improve trial efficiency and contribute to the sustainability of the clinical trial enterprise.

## Methods

### Study Design

This retrospective cohort study used data from REMAP-CAP; the design has been described previously.^3^ Reporting of the study followed the Strengthening the Reporting of Observational Studies in Epidemiology (STROBE) reporting guideline.^4^ Data were restricted to the European region (eTable 1 in Supplement 1) because these are governed by the same sponsor, the University Medical Center Utrecht (the Netherlands). We defined the pre–COVID-19 pandemic period as the start of REMAP-CAP on February 19, 2016, until March 10, 2020, and the COVID-19 pandemic period as March 11, 2020, until May 4, 2023 (as defined by the World Health Organization).^5^

### Data Collection

All data were centrally managed by the sponsor and additionally retrieved as needed from regional trial teams. Dates of first randomization per domain were derived from the electronic data capturing system. This EARL study does not fall under the scope of the Dutch Medical Research Involving Human Subjects Act and therefore did not require review by an accredited research ethics committee (REC) in the Netherlands or participant informed consent.

### EARL Procedures

The REMAP-CAP protocol has a modular structure of different elements, consisting of a core protocol that outlines the trial’s overarching structure and procedures, and domain-specific appendices (DSAs) that outline the specific interventions within each (treatment) domain. For background, a DSA is comparable to a traditional clinical trial protocol in the sense that it describes essential trial elements such as interventions and eligibility criteria, outcome measures, and safety end points tailored to the specific interventions. New domains can be added, and within domains, interventions can be added or removed through a new or updated DSA. Each DSA functions as a regulatory document requiring separate regulatory and ethical approval. Sites can participate in 1 or multiple DSAs simultaneously and in all or only some of the interventions within a DSA, depending on local availability and standard of care. Consequently, there is variation in participation in domains and interventions across hospital sites, both within and between countries.

During both periods, the applicable legislation for clinical trials in the European Union (EU) and European Economic Area (EEA) was based on the Clinical Trials Directive (CTD).^6,7^ In countries outside the EU and EEA, such as the UK, clinical trial legislation operates independently and is governed by national regulatory frameworks. The core protocol, applicable DSAs, and other (central) trial documents were submitted for review to national competent authorities (CAs) and RECs of each participating country separately. National laws and regulations required compliance with country-specific EARL requirements, such as relabeling of authorized marketed drugs. In February 2024, after this EARL study period, the trial transitioned to the EU Clinical Trial Regulation (CTR).^8^

### Outcome Measures

While DSAs are formally modular elements of the trial protocol, they will be referred to as protocols here to align with more common research terminology. Our outcomes included (1) site contract completion times, defined as the number of days between sending the first draft of the first clinical trial agreement or provision of resources to the study site until the agreement was fully signed by site and sponsor; (2) TTA per protocol (ie, DSA) submission, defined as the total time required to secure approval, measured as the number of days from submission (to either CA or REC, whichever was first) until approval by both CA and REC (a protocol submission could be part of a primary submission or a substantial amendment; if the submission was rejected and resubmitted, we counted from the first [original] submission date); and (3) time from regulatory and ethical approval to FPI, defined as the number of days between full approval of a protocol by both CA and REC until the first patient was randomized to that domain (FPI).

### Statistical Analysis

Because data were nonnormally distributed, descriptive statistics were reported as median and IQR or range. There was no formal statistical analysis plan, but comparisons were determined preanalysis based on key research questions. We analyzed the percentage reduction in median site contract completion time between the 2 periods separately for the UK and non-UK countries. Median TTA per protocol submission was calculated for both periods and compared between the UK and non-UK countries, and we assessed the 6 protocols with the largest variation in TTA. Time from approval to FPI was determined for the COVID-19 pandemic period only because the pre–COVID-19 pandemic period had relatively few new domains and FPIs and was compared between the UK and non-UK countries. Bootstrapping (10 000 resamples) was used to calculate 95% CIs using the BCa method for percent reduction and median differences. All statistical analyses and visualizations were performed using RStudio version 2024.09.0 (Posit) from November 2024 to March 2025.

## Results

**Figure 1.**
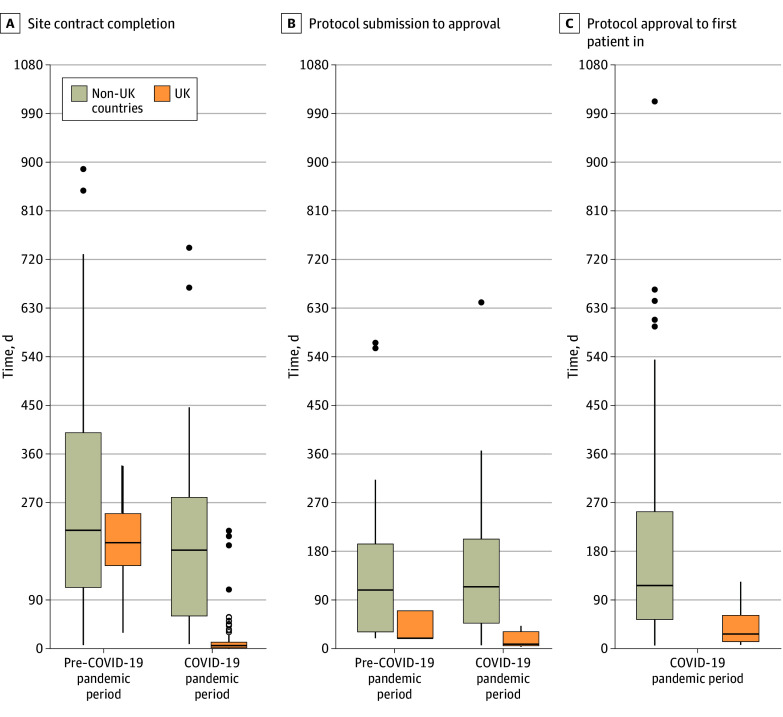
Site Contract Completion Time, Time to Approval, and Time From Approval to First Patient In The boxplot displays the distribution of data, with the end of the boxes representing the first quartile and third quartile (ie, the IQR) and the median represented by the middle line. Whiskers extend to the smallest and largest values within 1.5 times the IQR. Any values outside these lines are plotted as outliers. Two outliers for site contract completion time (1195 and 1733 days for pre–COVID-19 pandemic period and non-UK) were included in analyses but omitted from this plot for visibility reasons. See the Table for results by country.

The main outcomes by period and region are presented in Figure 1, providing an overview of key differences in speed between the UK and non-UK European countries. The Table illustrates the variability in timelines observed among the different countries.

**Table.** Site Contract Completion Time, Time to Approval, and Time to First Patient In by Country^a^

Country	Site contract completion time				Time to protocol approval^b^				Time from approval to FPI^c^	
	Pre–COVID-19 pandemic^d^		COVID-19 pandemic		Pre–COVID-19 pandemic		COVID-19 pandemic		COVID-19 pandemic	
	Sites, No.	Days, median (range)	Sites, No.	Days, median (range)	Approvals, No.	Days, median (range)	Approvals, No.	Days, median (range)	Implemented domains, No.	Days, median (range)
All countries	47	205 (6-1733)	210	13 (0-1195)	71	70 (19-567)	161	88 (3-642)	79	89 (5-1014)
All non-UK countries	32	224 (6-1733)	78	183 (8-1195)	64	109 (19-567)	144	115 (6-642)	65	116 (5-1014)
UK	15	196 (29-338)	132	5 (0-218)	7	19 (19-70)	17	8 (3-42)	14	26 (6-123)
Belgium	2	207 (58-355)	1	104	3	122 (122-122)	15	86 (40-135)	1	1014
Croatia	3	722 (73-889)	0	NA	3	557 (557-567)	4	47 (47-47)	0	NA
Czech Republic	0	NA	2	225 (106-344)	0	NA	8	294 (294-343)	6	377 (247-505)
Estonia	0	NA	1	183	0	NA	7	59 (59-120)	1	58
Finland	0	NA	2	48 (46-50)	0	NA	2	10 (9-11)	1	221
France	0	NA	23	68 (8-211)	0	NA	15	27 (6-76)	9	30 (14-609)
Germany	10	286 (89-731)	8	379 (32-743)	7	21 (21-182)	8	146 (88-146)	3	55 (55-56)
Hungary	1	849	0	NA	7	24 (24-95)	2	15 (15-15)	0	NA
Ireland	3	228 (205-409)	4	219 (60-303)	10	151 (45-254)	12	66 (16-252)	9	54 (7-281)
Israel	0	NA	1^e^	376	0	NA	NA^f^	NA	0	NA
Italy	0	NA	8	134 (78-370)	0	NA	12	148 (43-642)	10	186 (48-665)
The Netherlands	7	132 (6-219)	15	138 (26-669)	10	33 (19-135)	15	63 (25-164)	11	57 (5-180)
Portugal	2	276 (202-349)	1	404	7	125 (125-229)	2	55 (55-55)	0	NA
Romania	1	1733	1	1195	7	313 (284-313)	8	180 (63-214)	0	NA
Serbia	0	NA	3	406 (402-435)	0	NA	10	320 (320-320)	2	425 (253-597)
Slovenia	1	422	1	143	0	NA	8	367 (264-367)	5	152 (125-535)
Spain	2	145 (125-165)	6	285 (20-416)	10	57 (22-68)	8	123 (92-123)	6	125 (108-644)
Switzerland	0	NA	1	248	0	NA	8	203 (203-203)	1	195

Abbreviations: FPI, first patient in; NA, not applicable.

^a^ All values are presented as median (range). While median (IQR) was used in the text and figures for representing central dispersion, this table includes the full range to show the entire spread in durations.

^b^ In some countries, there were simultaneous submissions which were approved on the same date (ie, the median and range are identical).

^c^ Defined as the time between regulatory and ethical approval of a protocol (ie, domain-specific appendix) and the first patient randomized (FPI) to that domain (see eTable 2 in Supplement 1 for data by domain). Not all domain-specific appendices that were approved during the pandemic led to a patient randomization in the respective country.

^d^ Defined as agreements where negotiations began before March 11, 2020. These contracts may have been fully signed during the COVID-19 pandemic period.

^e^ Two additional sites were contracted, but the exact durations of contract negotiations could not be determined.

^f^ Three protocols were approved, but the exact review durations could not be determined.

### Site Contract Completion Time

During the study period, there were 257 fully signed first contracts with study sites for analysis. In the UK, median (IQR) contract completion times were 196 (154 to 250) days at 15 sites in the pre–COVID-19 pandemic period and 5 (1 to 11) days at 132 sites in the COVID-19 pandemic period, a 97% (95% CI, 95% to 98%) reduction (Figure 1). In non-UK countries, median (IQR) contract completion times were 224 (119 to 412) days at 32 sites in the pre–COVID-19 pandemic period and 183 (62 to 291) days at 78 sites in the COVID-19 pandemic period, a −18% (95% CI, −43% to 52%) difference.

### TTA

Primary submission of the trial occurred in 19 countries. The submission of 44 different interventions within 16 treatment domains, of which 24 interventions and 8 domains were specific to patients with suspected or proven COVID-19, resulted in 232 approved protocol submissions for analysis.

**Figure 2.**
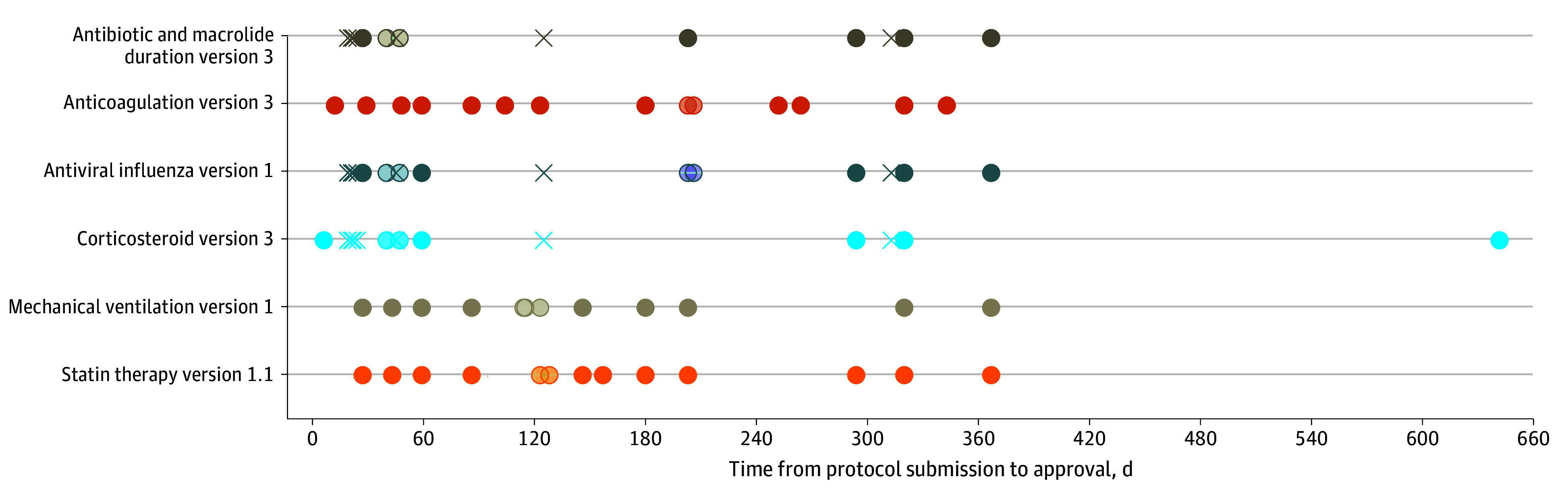
Variation in Time to Approval of Identical Protocols Across Different European Countries The 6 protocols with the largest variation in time to approval are displayed. Each data point represents time to approval in a different country. An X indicates pre–COVID-19 pandemic submission, while all others were submitted during the COVID-19 pandemic.

During the pre–COVID-19 pandemic period, median (IQR) TTA in the UK was 19 (19 to 70) days (7 approvals including 4 simultaneous approvals at 19 days) and 109 (31 to 194) days (64 approvals) in other countries (median difference, 90 days; 95% CI, 38 to 142 days) (Figure 1). During the COVID-19 pandemic, median (IQR) TTA was 8 (5 to 31) days (17 approvals) in the UK and 115 (47 to 103) days (144 approvals) in non-UK countries (median difference, 107 days; 95% CI, 76 to 123 days). There was substantial variation in TTA of an identical protocol between countries (Figure 2). The largest difference was for a corticosteroid protocol that was fully approved within 6 days in France and after 642 days in Italy (including the rejection and resubmission of an unchanged protocol).

### Time to FPI

Since the start of the trial, 208 sites in 16 countries (77 hospital wards and 201 intensive care units [some sites included both]) enrolled at least 1 patient. During the COVID-19 pandemic, the median (range) time from regulatory and ethical approval of a new domain to FPI across all countries was 89 (5 to 1014) days (Table and eTable 2 in Supplement 1 for data by domain and country). This period was, on average, 3 months longer in non-UK countries compared with the UK, with a median (range) of 26 (6 to 123) days in the UK and 116 (5 to 1014) days in non-UK countries (median difference, 90 days; 95% CI, 42 to 141 days) (Figure 1).

## Discussion

This cohort study quantified challenges in delivering RCTs. Key EARL hurdles included lengthy contract completion times for individual study sites and prolonged and variable TTA at the national level, which delayed the start of participant randomization, even during a public health emergency such as a pandemic.

Moreover, this study emphasizes the disparity in trial initiation between the UK and most other European countries. Trial endorsement and accelerated EARL procedures in the UK enabled rapid trial implementation in a large number of study sites. Importantly, the subsequent ability to enroll many patients in the UK enabled the fast identification of effective and noneffective treatments for patients with severe COVID-19, results which impacted patient care globally.^9,10,11,12,13,14,15,16,17,18^ We identified 3 actionable lessons from our analyses.

First, prior to the COVID-19 pandemic, the process of contracting sites was time consuming across all participating countries, with median durations of 224 days in non-UK European countries and 196 days in the UK. Despite having standardized contracts in place, in practice, contract deviations and negotiations still occurred in the UK. However, at the onset of the COVID-19 pandemic, the UK introduced a process for designating individual COVID-19 studies as having priority status, alongside REMAP-CAP being identified as 1 of 3 clinical trials supported by the Chief Medical Officer.^2^ This prioritization was designed to remove barriers for hospitals to participate, including a robust policy against adapting or negotiating, leading to a 97% reduction in median contract completion time to just 5 days. In contrast, traditional contract adaptations and negotiations continued in non-UK countries, without a large time reduction during the COVID-19 pandemic.

Second, times between submission and approval for identical protocols were highly variable between countries. Before and during the COVID-19 pandemic, the UK had a median TTA of 19 and 8 days, respectively. In contrast, median approval times across non-UK European countries were 109 days before and 115 days during the COVID-19 pandemic. Even though many countries implemented expedited processes, among the non-UK countries, only France achieved an average approval time of less than 1 month during the COVID-19 pandemic. Finland and Hungary also had average approval times of less than 1 month, but with only 2 submissions each, which makes it difficult to draw firm conclusions. In France, a law was passed to establish fast-track authorization for prioritized trials, and certain volunteer RECs even assessed COVID-19 study applications daily.^19^ The observation that most other countries had long review durations—possibly due to limited workforce and resource availability—raises concerns about the ability to effectively streamline approval processes during future pandemics. Interestingly, faster review processes did not seem to compromise the quality of the protocol assessments because the protocols underwent little to no changes, even during the lengthier review periods.

Even though the CTD^5^^7^ provided the regulatory framework for clinical trials in the EU and EEA, each country had discretion in how it was implemented within national legislation. The identified inconsistency in review durations for identical protocols across countries resulted from varying interpretations of trial regulations, informed consent procedures, medication handling, and clarification questions about the trial. The particularly large variation for the corticosteroid domain is explained by Italy, where the protocol was part of the trial’s primary submission, which was rejected, and where the (unchanged) protocol was resubmitted later. The CTR,^8^ effective since January 2022, introduced the centralization of clinical trial applications across the EU with predefined review timelines to harmonize and facilitate large multinational clinical trials. Submission under CTR may reduce inconsistencies in the future, although each member state retains authority over national requirements. Substantial regulatory and administrative burden has been reported by others.^20^ Furthermore, certain national requirements—such as those governing the relabeling of market-authorized drugs for critically ill patients—are still ambiguous. Of note, even procedural changes, like adding new trial sites or principal investigators, are considered substantial modifications, both under CTD and CTR, delaying site onboarding. In contrast, in the UK, adding new study sites is now considered a nonsubstantial amendment.^21^

Third, even after site contracts and regulatory and ethical approvals were achieved, delays in trial medication handling hampered the start of patient enrollment. This issue was particularly evident in the Netherlands because this was the only country where during the COVID-19 pandemic, full clinical trial relabeling was required for repurposed drugs such as simvastatin and aspirin, despite their established safety profiles and their administration in the trial by trained health care professionals. This process, which necessitated central pharmacy oversight and distribution, caused substantial delays and massive (public) resource consumption. In contrast, countries such as the UK, France, Spain, and Portugal waived clinical trial labeling requirements for repurposed drugs because of the public health emergency. Other countries required simplified labels for the same drugs, adding to the operational complexity. This variability in requirements between countries not only delayed the time between regulatory approval and FPI but also increased the overall burden and administrative workload for health care professionals and trial coordination in general. Whether relabeling market-authorized drugs truly enhances participant safety for trials that are limited to hospitalized patients remains debatable and reflects the discrepancy between the regulation of clinical research and clinical care.^22^

To our knowledge, this study is the first to identify and quantify EARL procedures, leveraging data from an ongoing multinational adaptive platform trial. Previous studies focused on a single country^23^ or assessed durations of primary submissions and first amendments during the COVID-19 pandemic, also revealing heterogeneity between countries.^24^ This study expands on this by analyzing multiple EARL procedures across numerous countries, both before and during a pandemic, for identical protocols from a single sponsor. While our analysis focuses on the European region, many of the challenges and lessons are likely applicable in other regions.^25^ The positive impact of streamlined and simplified processes in the UK during the COVID-19 pandemic on timelines and recruitment stands out. There is a need to move toward trial procedures that are fit for purpose, in particular in the context of public health emergencies.^26^ Addressing EARL hurdles with all stakeholders is key for ensuring that clinical trial systems are agile, efficient, and responsive when rapid action is required, and should be a central aspect of pandemic preparedness.

### Limitations

This study had several limitations. First, we analyzed TTA for protocols individually, even though multiple protocols could be submitted together as a single package. This approach was chosen due to the variability in how protocols were combined into submission packages across different countries. We consider this approach valid because the review duration per protocol is ultimately what impacted trial initiation for a new domain. Second, variability in approval time of identical submitted protocols was chosen as a proxy to quantify the variability in regulatory and ethical assessments between countries. A more in-depth, qualitative analysis of these assessments was beyond the scope of this paper. Third, the analysis on TTA intentionally did not differentiate between the relative response times of different parties because the primary objective was to quantify the full time needed to secure approvals as a reflection of total EARL process duration. Fourth, by analyzing time to first patient randomization rather than screening, the time needed to implement a new domain might be overestimated in countries where many patients were screened but found ineligible for randomization. However, because time between screening and randomization was generally short because of trial procedures, we do not expect this to significantly impact our conclusions. Lastly, due to small numbers for individual countries, data from non-UK countries were pooled for statistical comparisons, which may obscure differences in timelines between these countries. To address this, detailed data per country are provided in the main study.

## Conclusions

This cohort study found that EARL procedures were lengthy and variable between countries, with markedly faster processes in the UK. While robust processes are essential for patient safety and ethical conduct, current EARL procedures are often inefficient, delaying clinical trial initiation and consuming substantial resources. Specific actions to streamline EARL procedures without compromising quality or robustness include the adoption of standardized, harmonized, and nonmodifiable contracts and waiving relabeling requirements for repurposed drugs in in-hospital trials.

Variability and delays in EARL procedures can reduce the ability to efficiently conduct clinical trials, which in turn inhibits efforts to improve patient outcomes through robust, high-quality research, if they are not fit for purpose. Failing to address these challenges will limit our response capability during future public health emergencies, such as pandemics.

## Notes

Supplement 1. **eTable 1.** Definition of the European Region **eTable 2.** Time (Days) Between Regulatory and Ethical Approval to First Patient In During the COVID-19 Pandemic, by Domain and Country

Supplement 2.
Data Sharing Statement
