# Error in Figure

**DOI:** 10.1001/jamanetworkopen.2026.27624

**Published:** 2026-07-16

In the Original Investigation titled “Hurdles for the Delivery of Multinational Randomized Clinical Trials,”^1^ published July 2, 2025, there was an error in Figure 1. Panel C (protocol approval to first patient in) originally listed the comparison as the pre–COVID-19 pandemic period vs the COVID-19 period. The comparison was between UK and non-UK countries during the COVID-19 pandemic period only. This article has been corrected.^1^

## References

[1] van Hout D, Mouncey P, Harrison D, Bonten M, Derde L; REMAP-CAP European Regional Investigators. Hurdles for the delivery of multinational randomized clinical trials. JAMA Netw Open. 2025;8(7):e2518503. doi:10.1001/jamanetworkopen.2025.18503 40601320 PMC12223866

