# Supplementary material for: Hurdles for the Delivery of Multinational Randomized Clinical Trials
Source: JAMA Netw Open. 2025 Jul 2;8(7):e2518503. doi: 10.1001/jamanetworkopen.2025.18503 (PMC12223866; doi:10.1001/jamanetworkopen.2025.18503)
Supplement: Supplement 1. — eTable 1. Definition of the European Region eTable 2. Time (Days) Between Regulatory and Ethical Approval to First Patient In During the COVID-19 Pandemic, by Domain and Country [file jamanetwopen-e2518503-s001.pdf]

Supplemental Online Content

van Hout D, Mouncey P, Harrison D, Bonten M, Derde L; REMAP-CAP European Regional Investigators. Hurdles for the delivery of multinational randomized clinical trials. *JAMA Netw. Open*. 2025;8(7):e2518503. doi:10.1001/jamanetworkopen.2025.18503

eTable 1. Definition of the European Region

eTable 2. Time (Days) Between Regulatory and Ethical Approval to First Patient In During the COVID-19 Pandemic, by Domain and Country

This supplemental material has been provided by the authors to give readers additional information about their work.

© 2025 van Hout D et al. *JAMA Network Open*.

eTable 1. Definition of the European Region

The European region in REMAP-CAP comprises the 27 European Union (EU) member states, the United Kingdom and other countries in the European region as listed below (not all of which are participating).

The countries to which this region applies are:

• Austria<br>• Albania<br>• Belgium<br>• Bosnia and Herzegovina<br>• Bulgaria<br>• Croatia<br>• Cyprus<br>• Czech Republic<br>• Denmark<br>• Estonia<br>• Finland<br>• France<br>• Germany<br>• Greece<br>• Hungary<br>• Ireland<br>• Israel<br>• Italy<br>• Latvia	• Lithuania<br>• Luxembourg<br>• Malta<br>• Montenegro<br>• Netherlands<br>• Norway<br>• Poland<br>• Portugal<br>• Romania<br>• Serbia<br>• Slovakia<br>• Slovenia<br>• Spain<br>• Sweden<br>• Switzerland<br>• Turkey<br>• Ukraine<br>• United Kingdom

© 2025 van Hout D et al. *JAMA Network Open*.

eTable 2. Time (Days) Between Regulatory and Ethical Approval to First Patient In During the COVID-19 Pandemic[a], By Domain and Country.

Formal protocol title	Main characteristic	BE	CH	CZ	DE	EE	ES	FI	FR	IE	IT	NL	RS	SI	UK
Antibiotic V3	different antibiotic treatments	-	-	388	-	-	-	-	30	-	-	-	597[b]	125	-
Macrolide Duration V3	duration of macrolides	-	-	505[b]	-	-	-	-	30	-	-	-	-	191	-
Antiviral Influenza V1	oseltamivir	1014	-	366	-	-	-	-	609	-	665[b]	-	-	-	-
Corticosteroid V3	hydrocortisone	-	195[b]	505[b]	-	-	-	-	14	-	530[b]	-	-	152	-
COVID-19 IM V1	ifn-beta + anakinra	-	-	-	-	-	-	-	-	17	-	116	-	-	42
COVID-19 AV V1	lopinavir/ritonavir	-	-	-	-	-	-	-	-	46	-	36	-	-	15
COVID-19 AV V2	HCQ	-	-	-	-	-	-	-	-	-	-	-	-	-	6
COVID-19 AV V3	ivermectin	-	-	-	-	-	-	-	-	54	-	-	-	-	-
COVID-19 IM V2	IL-6 receptor antagonists	-	-	-	-	-	-	221	-	94	48	57	-	-	6
MV V1	protocolized MV strategy	-	-	-	-	-	644[b]	-	137	281	226[b]	-	253	535	-
COVID-19 Immunoglobulin V1	convalescent plasma	-	-	-	-	-	-	-	-	-	-	-	-	-	11
COVID-19 Therapeutic Anticoagulation V1	TAC	-	-	-	-	-	-	-	-	89	-	-	-	-	11
Vitamin C V1/V2[c]	vitamin C	-	-	-	56	58	108	-	-	89	51	53	-	125	123
COVID-19 Statin Therapy V1/ V1.1[c]	simvastatin	-	-	296	55	-	152	-	30	7	48	86	-	-	105
COVID-19 Antiplatelet V1	aspirin, P2Y12 inhibitors	-	-	-	55	-	-	-	30	-	48	68	-	-	18
COVID-19 ACE2RAS V1	ACEi, ARB	-	-	-	-	-	-	-	-	-	146	11	-	-	17
COVID-19 Therapeutic Anticoagulation V2	expansion to moderately ill patients	-	-	-	-	-	-	-	-	-	-	5	-	-	34
COVID-19 Antiplatelet V2	change in endpoint	-	-	-	-	-	133[b]	-	-	-	-	180	-	-	-
COVID-19 Immunoglobulin Therapy V3	high-titer convalescent plasma	-	-	-	-	-	-	-	-	-	-	65	-	-	102
COVID-19 Anticoagulation V3	strata based on prior TAC use	-	-	247	-	-	117	-	63	54	328	31	-	-	41
Cysteamine V1	cysteamine	-	-	-	-	-	-	-	-	-	-	-	-	-	67
Endothelial Modulation V1	enteral imatinib	-	-	-	-	-	116[b]	-	257[b]	-	306[b]	-	-	-	-

ACEi, ACE-inhibitors; ARB, angiotensin receptor blockers (i.e., angiotensin II receptor antagonists); AV, antiviral; FPI, first patient in; HCQ, hydroxychloroquine; ifn-beta, interferon beta-1a; IL-6, interleukin-6; IM, immune modulation; MV, mechanical ventilation; TAC, therapeutic anticoagulation

Country abbreviations: BE, Belgium; CH, Switzerland; CZ, Czech Republic; DE, Germany; EE, Estonia; ES, Spain; FI, Finland; FR, France; IE, Ireland; IT, Italy; NL, the Netherlands; RS, Serbia; SI, Slovenia; UK, United Kingdom

[a] Defined as the time between ethical and regulatory approval of a protocol and the first patient randomized to that domain. Domains for which the first regulatory or ethical submission date occurred during the COVID-19 pandemic as defined by the WHO were considered for this analysis (i.e., between 11 March 2020 and 5 May 2023).

[b] Date of FPI outside study period of the current EARL paper (i.e., after 5 May 2023), but submission during pandemic and therefore included in analyses.

[c] The submitted version may vary by country.

© 2025 van Hout D et al. *JAMA Network Open*.
