# Supplementary material for: Hurdles for the Delivery of Multinational Randomized Clinical Trials
Source: JAMA Netw Open. 2025 Jul 2;8(7):e2518503. doi: 10.1001/jamanetworkopen.2025.18503 (PMC12223866; doi:10.1001/jamanetworkopen.2025.18503)
Supplement: Supplement 2. — Data Sharing Statement [file jamanetwopen-e2518503-s002.pdf]

Data Sharing Statement

van Hout. Hurdles for the Delivery of Multinational Randomized Clinical Trials. *JAMA Netw Open*. Published July 02, 2025. doi:10.1001/jamanetworkopen.2025.18503

Data
Data available: Yes
Data types: Data (not involving human participants)
How to access data: eu.remapcap@umcutrecht.nl
When available: With publication

Supporting Documents
Document types: None

Additional Information
Who can access the data: Data will be available to researchers on request subject to sponsor restrictions.
Types of analyses: Research.
Mechanisms of data availability: After approval of a proposal, with investigator support.
